# The renoprotective potential of montelukast: a scoping review

**DOI:** 10.1097/MS9.0000000000002085

**Published:** 2024-04-22

**Authors:** Roham Sarmadian, Abolfazl Gilani, Saba Mehrtabar, Sona Mahrokhi Koushemehr, Zahra Hakimzadeh, Parsa Yousefichaijan

**Affiliations:** a Infectious Disease Research Center; b Department of Pediatrics, Arak University Of Medical Sciences, Arak; c Sina Trauma and Surgery Research Center; d School of Medicine, Tehran University of Medical Sciences, Tehran; e Neurosciences Research Center (NSRC); f Immunology Research Center, Tabriz University of Medical Sciences, Tabriz, East Azerbaijan, Iran

**Keywords:** acute kidney injury, chronic kidney disease, montelukast, nephrotoxicity, renal injury, renoprotection

## Abstract

**Introduction:**

Kidney damage can result from various factors, leading to structural and functional changes in the kidney. Acute kidney injury (AKI) refers to a sudden decline in kidney function, while chronic kidney disease involves a gradual deterioration lasting more than 3 months. Mechanisms of renal injury include impaired microcirculation, inflammation, and oxidative stress. Cysteinyl-leukotrienes (CysLTs) are inflammatory substances contributing to tissue damage. Montelukast, a leukotriene receptor antagonist, has shown potential renoprotective effects in experimental models of kidney injury.

**Methods:**

The authors conducted a scoping review using PubMed, Scopus, and Web of Science databases to identify relevant studies investigating the impact of montelukast on renal diseases. Articles published until 2022 were included and evaluated for quality. Data extraction and analysis were performed based on predetermined inclusion criteria.

**Results:**

The scoping review included 30 studies from 8 countries. Montelukast demonstrated therapeutic effects in various experimental models of nephrotoxicity and AKI induced by agents such as cisplatin, lipopolysaccharide, diclofenac, amikacin, *Escherichia coli*, cyclosporine, methotrexate, cobalt-60 gamma radiation, doxorubicin, and cadmium. Studies involving human subjects with nephrotic syndrome, pyelonephritis, and other renal diseases also reported positive outcomes with montelukast treatment. Montelukast exhibited anti-inflammatory, anti-apoptotic, antioxidant, and neutrophil-inhibiting properties, leading to improved kidney function and histopathological changes.

**Conclusions:**

Montelukast shows promise as a renoprotective medication, particularly in early-stage kidney injury. Its ability to mitigate inflammation, oxidative stress, and neutrophil infiltration contributes to its therapeutic effects. Further research is needed to explore the clinical applications and mechanisms underlying the renoprotective action of montelukast.

## Introduction

Highlights

- The review confirms that montelukast is a safe and effective choice for improving renal function, supported by the review of several research studies.
- Montelukast consistently exhibited therapeutic advantages in different types of kidney injuries, highlighting its diverse ability to protect the kidneys.
- Montelukast has been validated by clinical trials for its beneficial effects on renal disorders in humans, namely in terms of reduced corticosteroid usage and prevention of relapses.
- Understanding montelukast’s effects on acute kidney injury mechanisms helps to better understand its targeted intervention capabilities.

A normal kidney has about a million nephrons that cooperate to do its primary functions, which include maintaining the body’s overall fluid balance and pH, filtering waste from the blood, and hormonal functions that support the production of red blood cells, bone health, and blood pressure regulation^1^.

Renal damage can be described as changes in the structure or function of the kidney, even in the absence of primary changes in the glomerular filtration rate (GFR)^1^. It can be brought on by any stimuli that induce the loss of renal cells or changes in the structure of nephrons (includes the glomeruli, tubules, intrarenal blood vessels, and interstitium)^2^.

These stimuli can include infection, chemotherapeutic agents [cisplatin (CIS), methotrexate (MTX), and doxorubicin], immunosuppressant agents (cyclosporine), antibiotics (aminoglycosides), anti-inflammatories (diclofenac), metals (mercury and cadmium), and radiation^3–8^. The presentation of nephrotoxicity can range from an acute or chronic decrease in GFR to nephrotic syndrome and electrolytic disorders, which are linked to tubulopathies and glomerulopathies^8^. Acute or chronic deterioration in kidney function can lead to substantial morbidity and mortality^9^.

A sudden and often reversible decline in the kidney function, as indicated by an increase in creatinine or a decrease in urine volume, is known as an acute kidney injury (AKI)^10–12^. It is usually classified based on the location of the primary pathology as prerenal, intrarenal, and postrenal injury^13^. Intrarenal injury characterizes true renal injury with impairment to the main structures of the kidney, and it is mostly related to the release of vasoconstrictors from the renal afferent pathways^10,14^. Extrarenal diseases cause prerenal and postrenal injuries by impairing renal blood flow autoregulation and subsequent decrease in GFR^15^.

A main difference between acute and chronic renal disease is the rate and duration of renal function decline, with chronic kidney disease being defined as lasting longer than 3 months based on structural and functional abnormalities^16^.

Although there are numerous reasons for renal cell death, the pathophysiology is the same and is typically associated with an impairment in the microcirculation. This mismatch between the delivery of oxygen and nutrients to the nephrons and increased energy demands (increased oxidative stress, loss of ATP) leads to inflammation, vascular and tubular injury, and alteration in kidney biochemical parameters and oxidative stress markers^17–19^.

Cysteinyl-leukotrienes (CysLTs) are generated from arachidonic acid through 5-lipoxygenase pathway in response to cell activation and act on the CysLT1 and CysLT2 receptors^20^. They are proven to be potent chemotactic agents and inflammatory mediators that cause tissue injury by increasing the microvascular permeability^21,22^.

In recent years, previous studies have shown that montelukast, as a selective antagonist of leukotriene receptors (with antioxidant, anti-inflammatory, and anti-apoptotic properties), has the possible renoprotective effect in different experimental models of renal injuries induced by ischemia/reperfusion, sepsis, burn, and nephrotoxic agents^23–31^. It also improves GFR in a rhabdomyolysis-induced acute renal failure (ARF) model and other kidney dysfunction disorders in rats^32,33^.

## Materials and methods

### Search strategy and eligibility criteria

This scoping review was conducted on recent advances in the evaluation of montelukast in renal diseases. For this, a thorough literature search was carried out electronically. The PubMed, Scopus, and Web of Science databases were searched using MeSH entry terms mixed with Boolean phrases “AND” or “OR”. The terms were searched for “Kidney”, “Renal”, “Nephrotic”, “Nephritic”, “Azotemia”, “Pyelonephritis”, “Urine”, and “Montelukast” in research titles or abstracts. Two researchers conducted separate searches within databases and assessed the quality of pertinent research. Any disagreements during this process were resolved via collaborative negotiations.

All database articles that were published until 2022 were included in the study. Duplications were discarded. Explicit representation of the included literature and summarization of the available insights regarding the topic of interest were done following the Preferred Reporting Items for Scoping Reviews (PRISMA2020) checklist^34,35^. To assess the methodological quality and reporting standards of our review, we employed the AMSTAR2 (A Measurement Tool to Assess Reviews 2) tool, which comprises 16 items designed to evaluate key aspects of systematic reviews. Two independent reviewers conducted the assessment and resolved any disagreements through discussion and consensus. Our review demonstrated a moderate level of compliance with the AMSTAR2 criteria.

### Data collection

**Figure 1:**
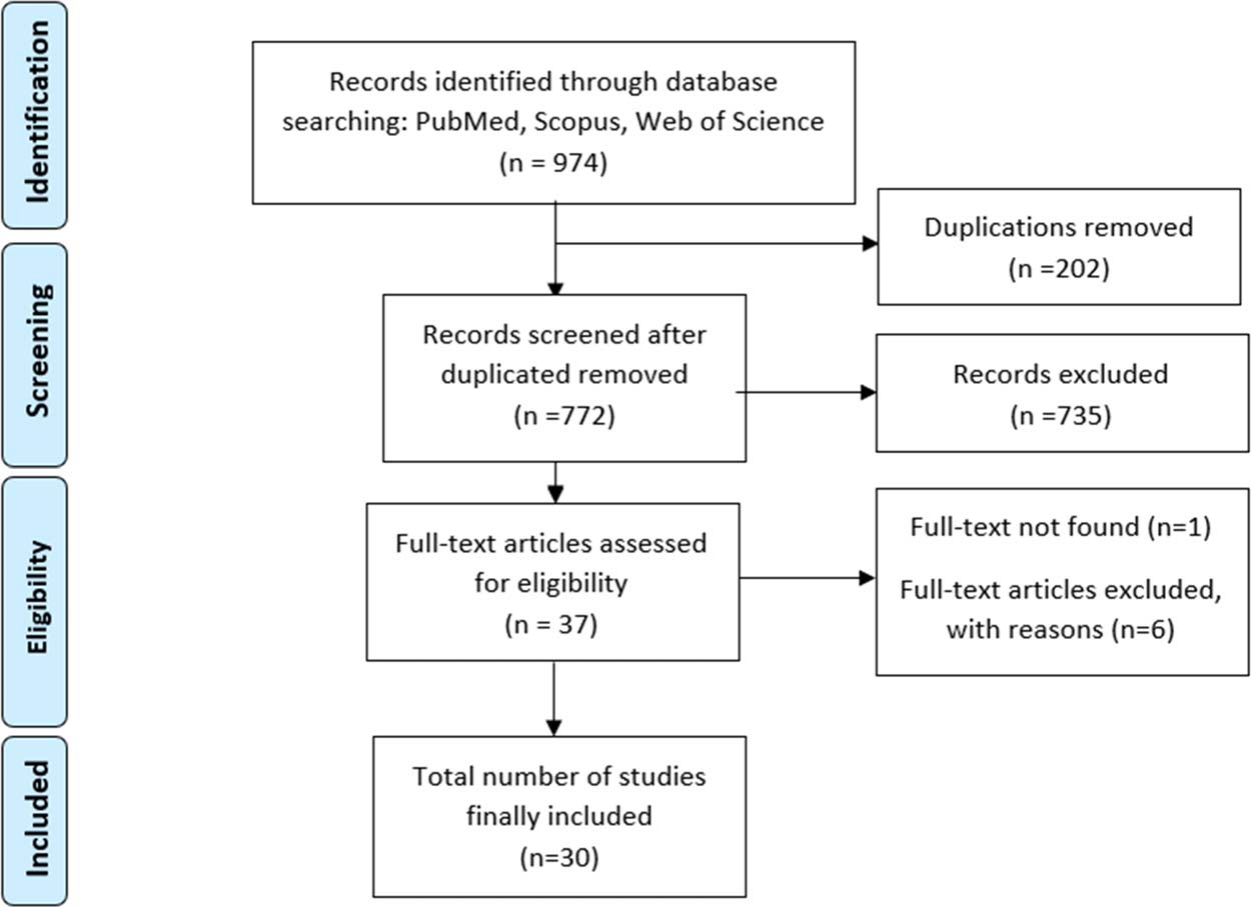
PRISMA flowchart: selection process and number of records at each stage.

The basic search resulted in 974 articles. The articles retrieved from the search were first imported into Endnote software. Any duplicate articles were identified and removed at that stage. The titles and abstracts of the remaining unique articles were then screened. Non-English articles, reviews, and meta-analyses were deemed ineligible for inclusion in the studies. Furthermore, meeting abstracts, case reports, letters to the editor, and editorial comments were also excluded from the analysis. Articles deemed irrelevant or unrelated based on their titles and abstracts were also excluded. The full texts of the remaining potentially relevant articles were reviewed next. Following that full-text review, any articles that were ultimately found to be unrelated were excluded. Finally, the desired information was extracted just from the remaining articles that were determined to be relevant. Figure 1 depicts the number of records at each stage.

### Data extraction

Two investigators autonomously gathered data from studies that met the predetermined eligibility criteria and categorized them according to the topic and its implications. In instances of discord, a consensus was achieved through deliberations involving a third reviewer.

The data extraction encompassed key elements such as the publication year, primary author, study design, type of renal disease, species (rat or human), and the ultimate conclusion.

## Results

The search strategy recovered 772 records after duplication; of these, 37 were found potentially eligible to be analyzed in our systematic review. At the end, 30 studies across 8 countries were included in this review. Of these 30 included records, all studies were randomized control trials (RCTs) with the exception of 2 case–control studies; 23 trials were performed on rats only, 1 on both human and rats, and 6 on only humans.

The information obtained from each of the extracted studies is displayed in Table 1. The effect of montelukast on CIS-induced kidney damage was examined in six studies^33,36–40,^ of which all except one^25^ showed that montelukast had therapeutic effects on CIS-induced nephrotoxicity by its anti-inflammatory and anti-apoptotic effects, but as mentioned above, the results of one study showed that montelukast had no protective effect on renal damage caused by CIS.

**Table 1:** Results obtained from the extracted articles

Reference number	Year	Country	Kind of study	Kind of renal disease	Rat/human	Conclusions
^24^	2014	Egypt	Randomized control trial (RCT)	Endotoxemia induced by LPS.	Rats	MNT has lung and renoprotective effects against the inflammatory process during endotoxemia because of its antioxidant and/or anti-inflammatory properties.
^30^	2018	Iraq	RCT	Acute kidney injury induced by diclofenac.	Rats	MNT has a protective effect against diclofenac-induced acute kidney damage through its effect on kidney biochemical parameters and oxidative stress markers.
^44^	2021	Egypt	RCT	Neonatal organ toxicity induced by maternal exposure to silver nanoparticles (AgNPs).	Rats	Montelukast antagonized the biochemical and histopathological changes occurred in kidneys and bones by antioxidant, anti-apoptotic, and anti-inflammatory actions with a possible role for EGF (epidermal growth factor).
^31^	2012	Turkey	RCT	Amikacin-induced kidney damage.	Rats	Montelukast treatment reduces the amikacin-induced kidney damage by reducing the expression of apoptotic cells.
^33^	2012	Egypt	RCT	Nephrotoxicity induced by cisplatin (CIS).	Rats	Montelukast probably antagonizes the constrictor effect of ACh on the urinary bladder and protects it from hypersensitivity to ACh induced by CIS treatment.
^36^	2017	Egypt	RCT	Nephrotoxicity induced by CIS.	Rats	Montelukast guards against CIS-induced nephrotoxicity via anti-inflammatory and anti-apoptotic properties.
^45^	2020	Japan	Case–control	Minimal change nephrotic syndrome (MCNS)	Humans	The addition of cetirizine and montelukast treatment for MCNS patients with prolonged disease duration concomitant with allergic disorders was effective in reducing daily corticosteroid dosage.
^41^	2007	Turkey	RCT	*Escherichia coli*-induced pyelonephritis.	Rats	It seems likely that montelukast protects kidney tissue by inhibiting neutrophil infiltration, balancing oxidant–antioxidant status, and regulating the generation of inflammatory mediators.
^32^	2012	Egypt	RCT	Intramuscular glycerol-induced rhabdomyolysis.	Rats	Montelukast abrogated functional and structural renal damage via ameliorating renal oxidative stress and modulation of systemic cytokines and apoptotic factors production.
^27^	2005	Turkey	RCT	Thermal trauma (burning with hot water)-induced remote organ and skin injuries.	Rats	Montelukast treatment reversed all biochemical indices, histopathological alterations induced by thermal trauma and possesses an anti-inflammatory effect on burn-induced damage in remote organs and protects against oxidative organ damage by a neutrophil-dependent mechanism.
^51^	2015	Turkey	RCT	The left ureter of the rats was sutured with 4-zero polyglactin sutures.	Rats	Montelukast and *N*-acetylcysteine have a protective effect against obstructive damage of the kidney.
^28^	2008	Turkey	RCT	Cyclosporine-induced nephrotoxicity.	Rats	The administration of montelukast, an LT receptor blocker, may prevent CsA-induced nephrotoxicity.
^77^	2011	Turkey	RCT	Cecal ligation and puncture-induced tissue injury of vital organs by inhibition of the proinflammatory cytokine response.	Rats	The lung and kidney tissues were the most protected by MLK under sepsis conditions. We can suggest that MLK reverses the systemic inflammatory reaction to polymicrobial sepsis and thereby reduces multiple organ failure.
^29^	2014	Egypt	RCT	Methotrexate-induced kidney damage in rats.	Rats	Montelukast significantly reduced the toxic effects of MTX as indicated from normalization of kidney-specific parameters, oxidative stress, and inflammatory mediators.
^76^	2020	Iran	RCT	Nephrotoxicity induced by whole‐body irradiation was performed with a cobalt‐60 gamma radiation source.	Rats	Montelukast has a potential role to be used as a renal protective agent against gamma radiation in radiotherapy.
^42^	2021	Pakistan	Case–Control	Relapses in childhood idiopathic steroid-sensitive nephrotic syndrome (SSNS).	Humans (2–6 years)	Montelukast showed significant efficacy in preventing relapse rate in children with SSNS during 1 year of follow-up.
^37^	2014	Romania	RCT	Cisplatin-induced experimental acute renal failure.	Rats	The obtained results showed that Montelukast at a dose of 2 mg/kbw had no protective effect in CIS-induced experimental acute renal failure.
^25^	2019	Turkey	RCT	Doxorubin (DOX)-induced acute kidney damage.	Rats	ML treatment after DOX injection caused therapeutic effects against DOX-induced kidney damage. Thence, ML treatment is of some clinical properties for oxidative stress damage in kidney tissues.
^50^	2006	Turkey	RCT	Rats were unilaterally nephrectomized and subjected to 45 min of renal pedicle occlusion followed by 6 h of reperfusion.	Rats	MNT reversed ischemia/reperfusion-induced oxidant responses, improved microscopic damage and renal function, and protects kidney tissue by inhibiting neutrophil infiltration, balancing oxidant–antioxidant status, and regulating generation of inflammatory mediators.
^38^	2012	Turkey	RCT	Cisplatin-induced acute renal damage.	Rats	Montelukast treatment after CIS injection exerted therapeutic effects against CIS-induced acute kidney damage.
^39^	2018	Egypt	RCT	Cisplatin-induced nephrotoxicity.	Rats	Zileuton abrogates CIS nephrotoxicity in rats probably via the inhibition of detrimental actions of 5-LOX products, thus favorably affecting renal oxidative/inflammatory/caspase-3 axis.
^26^	Turkey	2020	RCT	The right renal pedicle was occluded for 45 min to induce ischemia and then reperfused for 6 h.	Rat	The mean pathological scores of montelukast were significantly lower than those of the placebo group. Also in biochemical examination, significant differences were found. The administration of montelukast sodium was seen to have a nephroprotective effect against the development of renal damage associated with warm renal ischemia.
^78^	Thailand	2013	RCT	Renal cyst	Dog [Madine Darby canine kidney (MDCK)] cells	Montelukast (50 mM) reduced percent of cyst colonies.
^40^	Egypt	2018	RCT	Cisplatin-induced acute renal damage.	Rat	Reversed the CIS-induced nephrotoxicity.
^46^	Egypt	2016	RCT	Steroid-dependent nephrotic syndrome.	Humans	The Montelukast group showed a significant decrease in serum creatinine and a significant increase in diastolic blood pressure and protein/creatinine ratio and a marked decrease in plasma LTC4/D4/E4 compared to the LDS group.
^21^	Turkey	2007	RCT	Chronic renal failure	Rat	Montelukast reduces CRF-induced neutrophil accumulation, oxidative injury, and renal dysfunction. These protective effects of montelukast on chronic renal failure-induced injury can be attributed to its ability to inhibit neutrophil infiltration and apoptosis, to balance oxidant–antioxidant status and to regulate the generation of proinflammatory mediators.
^47^	Iran	2021	RCT	Nephrotic syndrome	Human (pediatric)	Recovery rate was higher in the intervention group, but the difference was not statistically significant.
^48^	Iran	2019	RCT	Pyelonephritis	Human (pediatric)	Montelukast leads to rapid improvement of clinical manifestations in children with pyelonephritis and may be used as an effective auxiliary treatment in these patients.
^49^	Turkey	2014	RCT	Renal damage after unilateral ureteral obstruction in rats.	Rats	Montelukast prevents kidney damage with antioxidant effect, independently of NO.
^43^	Iran	2021	RCT (*in vivo* and *in vitro*)	Renal damage due to cadmium toxicity.	Both	The antioxidant properties of MNT can be considered as one of its protective mechanisms against cadmium toxicity. Decrease in the oxidative stress indices and increase in the antioxidant capacity.

ACh indicates Acetylcholine; LDS, low-dose steroid; MNT, montelukast.

We also examined several RCTs about the effect of montelukast in nephrotoxicity or acute kidney damages in rats induced by lipopolysaccharide (LPS)^24^, diclofenac^30^, amikacin^31^, *Escherichia coli*^41^, cyclosporine^28^, MTX^29^, cobalt-60 gamma radiation sources^42^, doxorubin^25^, and cadmium^43,^ and all studies resulted in montelukast having therapeutic effects by its anti-inflammatory, anti-apoptic, and antioxidant effects. It has been shown in multiple studies that montelukast also has an effect on inhibiting neutrophil infiltration.

One study that was done about the effects of montelukast on organ damage induced by neonatal exposure to silver nanoparticles (AgNPs) also resulted in montelukast antagonizing the biochemical and histopathological changes in kidneys and bones^44^.

Studies examining montelukast effect on humans with minimal change nephrotic syndrome (MCNS)^45^, steroid-sensitive nephrotic syndrome (SSNS)^42,46^, nephrotic syndrome^47^, and pyelonephritis^48^ had similar results and showed montelukast having great effects on decreasing corticosteroid dosage in treatment^45^, preventing relapse rate in children with SSNS^42^, a significant decrease in serum creatinine, and a significant increase in diastolic blood pressure and protein/creatinine ratio^46^, increasing the improvement rate of clinical manifestations in children with pyelonephritis^49^, with one exception of montelukast increasing the recovery rate but not statistically significant^48^.

By examining studies about montelukast effects on kidney damage caused by ischemia and reperfusion in rats^26,50,^ results showed montelukast having nephroprotective effects on kidney tissue and improving microscopic damage caused by ischemia by inhibiting neutrophil infiltration and regulating inflammatory and oxidative mediators.

Montelukast has been shown to have a significant protective effect against kidney damage caused by obstruction^49,51^.

One of the studies examined in this review also showed montelukast being useful in reversing histopathological and biochemical alterations caused by thermal trauma with anti-inflammatory and neutrophil-dependent mechanisms^27^.

Montelukast has also been shown to be effective in abrogating functional and structural renal damage caused by rhabdomyolysis induced by intramuscular glycerol by its antioxidant effects and modulating systemic cytokines and apoptotic factors^32^.

## Discussion

### Summary of the main findings

Following is a description of the results of the scoping review:

As described previously, studies show that montelukast has the potential to be used as a renal protective agent against: gamma radiation in radiotherapy, diclofenac-induced acute kidney damage, sepsis-induced degenerative changes, aminoglycosides nephrotoxicity, MTX/CIS-induced kidney damage, chronic cyclosporine (CsA)-induced nephrotoxicity, obstructive damage of the kidney, burn-induced damage, ischemia/reperfusion damage, pyelonephritis, rhabdomyolysis-induced ARF, and neonatal organ toxicity induced by maternal exposure to AgNPs. And it also showed significant efficacy in preventing relapse rate in children with SSNS.

AKI is a condition characterized by sudden renal function impairment. AKI causes electrolyte abnormalities and decreased urine production due to a reversible increase in nitrogenous waste products and serum creatinine content^52^. AKI has been documented in numerous nations. Moreover, it is associated with increased adult and juvenile mortality and morbidity. Furthermore, the diseases may progress to chronic renal failure^53^. AKI is becoming a significant public health concern. As the patient population ages in developed nations, it is anticipated that the incidence of AKI will increase proportionally^30,54^.

Leukotrienes induce immune-mediated injury in kidneys^42,55^. Recent studies have implicated elevated interleukin-13 (IL-13), a leukotriene, in the injury of podocytes, which induces minimal change like nephropathy. Downregulation of nephrin, podocin, and dystroglycan, which are essential for maintaining the integrity of slit diaphragms (SDs), is caused by IL-13 overexpression. IL-13 was also found to have an effect on ZO-1 proteins, resulting in the breakdown of the glomerular filtration barrier and the development of proteinuria in MCNS^42,56–58^.

In the study by Park *et al*.^55^, montelukast was shown to reverse the dysfunction of human podocytes caused by IL-13.

Montelukast is an anti-inflammatory medication with antioxidant properties that interferes directly with leukotriene reception (leukotriene receptor antagonist)^59^. CysLTs, specifically LTC4, LTD4, and LTE4, are potent proinflammatory lipid mediators secreted by eosinophils, mast cells, monocytes, and macrophages^60^. It has been shown that both leukotriene pathway modifiers and leukotriene receptor antagonists are efficacious in treating oxidative renal damage and renal ischemia/reperfusion injury^29,50^. Moreover, MLK was reported to decrease elevated TNF-α levels induced by oxidative renal damage in pyelonephritic rats^41^.

Overproduction of reactive oxygen radicals and neutrophil infiltration are significant causes of MTX-related renal toxicity^61,62^. Through a neutrophil-dependent mechanism, MLK has an anti-inflammatory effect against oxidative injury^63^. Consequently, MLK has a protective effect against renal injury induced by MTX due to its ability to inhibit neutrophil infiltration and to modulate the production of inflammatory mediators, in addition to its significant antioxidant potential^29^.

MTX inhibits the remethylation of homocysteine and causes both acute and chronic homocysteine elevations. Rapid auto-oxidation of homocysteine in plasma generates reactive oxygen species, such as superoxide and hydrogen peroxide radicals^64^. In addition, MTX decreases intracellular NADPH levels, which is required to maintain the reduced state of glutathione (GSH), resulting in a decrease in this essential cytosolic antioxidant substance^29^.

GSH is a hydroxyl radical and singlet oxygen scavenger. Abdel-Raheem *et al*.^29^ came to the conclusion that MTX caused a decrease in GSH levels and that MLK prevented the MTX-induced decline in GSH content and restored it to normal levels.

CIS endangers the kidney by decreasing GFR by activating angiotensin-converting enzyme to produce angiotensin II, which then releases ET-1 and CyLT^36,65^. Montelukast via barring CysTR1 inhibits the actions of renal vasoconstrictors LTD4 and LTC4 to improve GFR serological markers^66^.

Gad *et al*. revealed that MLK protected CIS-induced nephrotoxicity by inhibiting ET-1, MCP-1, TNF-α, and NO, confirming its anti-apoptotic activity and reducing anti-neoplastic-mediated HO-1 rise. MLK also significantly decreased kidney Casp-3 reactivity in CIS-induced apoptosis^36^.

Unlike the previous investigation, Teslariu *et al*. found no protective effect of MLK at 2 mg/kbw for 10 days in CIS-induced experimental ARF. Both trials used 5 mg/kg CIS, whereas Gad *et al*.’s research used 10 and 20 mg/kg/day MLK 5 days before and after single CIS^37^.

Nephrotoxicity is the most significant clinical complication of aminoglycosides treatment. The nephrotoxic effect of aminoglycosides is revealed by tubular necrosis in the absence of extensive glomerular morphological alterations. Generation of reactive oxygen radical species (ROS) is thought to be a second mechanism of this toxicity^31,67^.

The mechanism(s) of amikacin-induced renal injury are multifactorial and appear to involve free radical damage and inflammatory responses^68,69^.

In the study by Kose *et al*.^31^, one of the most significant findings was that amikacin increased the levels of malondialdehyde (MDA) and myeloperoxidase (MPO) in the amikacin-treated group. In addition, an increase in IL-1β levels in the same group indicates that amikacin was responsible for the inflammation and oxidative injury. The administration of montelukast after amikacin substantially decreased MPO levels. In contrast, montelukast treatment prior to amikacin administration did not noticeably alter the levels of MDA and MPO.

Gentamicin is taken up by endocytosis and accumulates in the lysosomes of the proximal tubules, resulting in lysosomal membrane rupture and tubular cell death^70^.

MLK inhibits megalin receptor expression/endocytic function, which uptakes and accumulates gentamicin in proximal tubular cells, reducing gentamicin nephrotoxicity, according to Azouz *et al*.^71^. MLK downregulates ClC-5, which regulates megalin endocytic function. MLK treatment lowers renal cell apoptosis and enhances kidney function without altering gentamicin antibacterial action.

In the case of radiotherapy for cancer, particularly in the case of abdominal tumors, renal function is significantly compromised during the examination, leading to undesirable side effects. Free radicals and reactive oxygen molecules generated by activated neutrophils, monocytes, and other cells during inflammatory processes appear to contribute to kidney injury^41,72^.

Multiple investigations have demonstrated that exposure to ionizing radiation (IR) can deplete GSH levels. Therefore, the replenishment of GSH in the kidneys, the reversal of elevated MPO activity, and the maintenance of oxidant–antioxidant balance can be regarded as the primary mechanisms by which MLK prevents kidney injury against IR in radiotherapy^73^,^74^. Also, the inhibition of nuclear factor kappa B activation is a crucial mechanism demonstrating MLK’s anti-inflammatory properties^75^.

MLK has the potential to be used as a renal protective agent against gamma radiation in radiotherapy, per the findings of Hormati *et al*.^76^. A higher level of MDA was detected in IR-treated rats, which is a result of the oxidative stress induced by IR in the kidney. However, MLK treatment reduced the MDA level. Administration of MLK prior to IR resulted in a concentration-dependent decrease in serum creatinine and urea levels, compared to rats treated with IR alone. The greatest reduction was seen in animals treated with MLK (10 mg/kg) and IR^76^.

Sahib *et al*. conducted a study to evaluate the protective effect of montelukast against the renal injury caused by diclofenac in rats. In comparison to diclofenac, the group treated with diclofenac plus montelukast had significantly decreased serum MDA levels and increased GSH levels. The diclofenac-treated group’s renal tissues were associated with varying degrees of kidney injury. The montelukast-treated group exhibited moderate accumulation of proteinaceous deposits in the renal tubular lumen, mild congestion, and no tubular necrosis. In comparison to the diclofenac-treated group, the diclofenac-plus-montelukast-treated group had substantially lower levels of serum urea and creatinine^30^.

Khodir *et al*. examined montelukast effects on lung and kidney damage in LPS-induced systemic inflammatory response. In this investigation, LPS administration enhanced lipid peroxidation and decreased endogenous antioxidant GSH in lung and kidney tissues, indicating substantial oxidative damage. Serum lactate dehydrogenase levels showed widespread tissue damage. MLK eliminated sepsis-induced oxidative damage and tissue inflammation. MLK also protected lung and kidney tissues against sepsis-induced degeneration, as shown by tissue morphology^24^.

Helmy *et al*. produced rhabdomyolysis in rats with intramuscular glycerol to test montelukast’s renoprotective effects. Rhabdomyolysis caused kidney structural (tubular necrosis) and functional (elevated serum creatinine, urea, and phosphate) changes, as well as oxidative stress, apoptotic factors (Fas), and cytokines (TNF-α, TGF-β1, and IL-10). Montelukast reduced rhabdomyolysis-related structural and functional damage by modulating cytokines and Fas production and enhancing antioxidant capacity. CysLT1 receptor inhibition might prevent rhabdomyolysis-induced ARF by modulating cytokines and correcting antioxidant profile abnormalities. It emphasizes the importance of treating traumatized individuals with effective renoprotective treatment like montelukast^32^.

In general, we argue that MLK is a safe and efficacious therapeutic intervention for enhancing renal function and reducing pathological remodeling in AKI. It is essential to personalize treatment to the specific necessities and disease stage of each patient.

In this review, we used the PRISMA checklist to figure out how well the studies were done from a scientific point of view. All of the RCT studies randomly assigned treatment or control groups and reported that they met legal standards. This shows that these studies had a good research design and were ethical. All included studies were published in credible peer-reviewed journals with credible manuscript verification and review procedures.

### Limitations

To minimize bias, we applied standard scoping review techniques and included all registered trials into the synthesis of this review. However, this review has several limitations.

Some limitations can be described as follows: We only extracted English-language studies, so research reports written in other languages were disregarded. In addition, conference papers, unpublished papers, or locally published papers were not included. Moreover, various durations and doses of MLK were utilized in the investigations. By limiting the search to ClinicalTrials.gov, it is possible that trials registered in other registries were overlooked. Most of the retrieved studies were early, with few reporting phase 3 outcomes. Commercial interest prevented many trials from publishing clinical data, which could have biased reporting. Finally, studies were done in diverse disease entities with different outcome measures examined at different time periods and varied dosage methods and combination therapies, making analyses difficult.

## Conclusions

On the basis of these findings and those that have previously described the pathophysiology of AKI, we propose that MLK can be used as a renoprotective drug in the early phases of kidney injury.

## Ethical approval

Ethical issues (including plagiarism, data fabrication, and double publication) have been completely observed by the authors. Our institution does not require ethical approval for reviews.

## Consent

Informed consent was not required for this review.

## Sources of funding

This research did not receive any funding.

## Author contribution

Conceptualization: A.G. and P.Y.; methodology: R.S. and A.G.; validation: S.M. and S.M.K.; formal analysis: not applicable; investigation: S.M., S.M.K., and R.S.; data curation and visualization: Z.H.; original draft preparation: S.M., S.M.K., Z.H., A.G., and R.S.; review and editing: R.S. and P.Y.; supervision: A.G.; and project administration: R.S.

## Conflicts of interest disclosure

The authors disclose no conflicts of interest.

## Research registration unique identifying number (UIN)

Not applicable.

## Guarantor

Abolfazl Gilani and Roham Sarmadian.

## Data availability statement

Data will be provided by the corresponding author on request.

## Provenance and peer review

Not commissioned, externally peer-reviewed.

## Consent for publication

Not applicable.
